# Soluble RAGE in COPD, with or without coexisting obstructive sleep apnoea

**DOI:** 10.1186/s12931-022-02092-9

**Published:** 2022-06-21

**Authors:** Marta Marin-Oto, David Sanz-Rubio, Fernando Santamaría-Martos, Ivan Benitez, Ana L. Simon, Marta Forner, Pablo Cubero, Ana Gil, Manuel Sanchez-de-laTorre, Ferran Barbe, José M. Marin

**Affiliations:** 1 Translational Research Unit, Aragón Health Research Institute, Zaragoza, Spain; 2 Respiratory Department, Hospital Arnau de Vilanova, IRB-Lleida, Lleida, Spain; 3 CIBER Enfermedades Respiratorias, Instituto Salud Carlos III, Madrid, Spain; 4 Respiratory Service, Hospital Universitario Miguel Servet and Department of Medicine, University of Zaragoza, Zaragoza, Spain

**Keywords:** sRAGE, Smokers, Chronic obstructive pulmonary disease, Obstructive sleep apnoea

## Abstract

**Background:**

Hypoxia can reduce the levels of soluble receptor for advanced glycation end-products (sRAGE), a new anti-inflammatory biomarker of COPD. We assessed sRAGE in patients with hypoxia-related diseases such as COPD, OSA and OSA-COPD overlap.

**Methods:**

Plasma levels of sRAGE were measured in 317 subjects at baseline (57 heathy nonsmokers [HNS], 84 healthy smokers [HS], 79 OSA, 62 COPD and 35 OSA-COPD overlap patients) and in 294 subjects after one year of follow-up (50 HNS, 74 HS, 77 OSA, 60 COPD and 33 overlap).

**Results:**

After adjusting for age, sex, smoking status and body mass index, sRAGE levels showed a reduction in OSA (− 12.5%, *p* = 0.005), COPD (− 14.8%, *p* < 0.001) and OSA-COPD overlap (− 12.3%, *p* = 0.02) compared with HNS. There were no differences when comparing sRAGE plasma levels between overlap patients and those with OSA or COPD alone. At follow-up, sRAGE levels did not change significantly in healthy subjects, COPD and OSA or OSA-COPD overlap nontreated with continuous positive airway pressure (CPAP). Moreover, in patients with OSA and OSA-COPD overlap who were treated with CPAP, sRAGE increased significantly.

**Conclusions:**

The levels of sRAGE are reduced in COPD and OSA. Treatment with CPAP appears to improve sRAGE levels in patients with OSA who also had COPD.

**Supplementary Information:**

The online version contains supplementary material available at 10.1186/s12931-022-02092-9.

## Background

Chronic obstructive pulmonary disease (COPD) is characterized by airflow obstruction and persistent airway inflammation due to the inhalation of noxious gases such as cigarette smoke [1], whereas obstructive sleep apnoea (OSA) is characterized by periodic collapse of the upper airway during sleep [2]. COPD and OSA are frequent diseases affecting more than 10% of the adult population each [1, 3]. The term ‘‘overlap syndrome’’ was introduced to describe the association of both conditions in a single patient. Patients with “overlap syndrome showed an increased risk of death and hospitalization [4, 5].

One of the main common consequences of these diseases is local and systemic hypoxia [5–7]. Systemic inflammation and oxidative stress develop because of chronic hypoxia [6] and in response to repetitive intermittent hypoxia during sleep in OSA [7]. Both COPD and OSA have been associated with an increased risk for cardiovascular morbidity and mortality, and it is possible that elevated systemic inflammation and oxidative stress could play an important role as intermediary mechanisms. sRAGE, the soluble isoform of the receptor for advanced glycation end products (RAGE), prevents AGE-enhanced MAP kinase activity, generates oxidative stress and inhibits the activation of proinflammatory signalling cascades [8]. Some studies suggest that sRAGE is protective against proinflammatory conditions and that RAGE could be a potential therapeutic target in chronic inflammatory diseases [9]. Reduced levels of sRAGE are related to both an increased risk of cardiovascular events [10]. The role of sRAGE in the pathophysiology of COPD has not been elucidated. Large COPD cohorts such as ECLIPSE, COPDGene, or SPIROMICS have reported that sRAGE is the best biomarker associated with airflow obstruction and emphysema [11–13], particularly centroacinar emphysema [14]. However, its association with emphysema and COPD progression are inconsistent in these cohorts [15]. The role of sRAGE in OSA has not yet been studied.

Both COPD and OSA are two diseases where hypoxia plays a crucial pathogenic role, contributing to inflammation and oxidative stress; therefore, these conditions may be associated with a lower level of sRAGE. We hypothesized that there is a reduction in plasma levels of sRAGE in OSA and COPD, with an additive decreasing effect in those patients with both conditions (overlap syndrome). The aim of the present study was to assess the plasma levels of sRAGE in OSA, COPD and OSA-COPD overlap patients and its relationship with disease severity variables such as lung function and hypoxemia. Afterwards, we assessed the effect of treatment with continuous positive airway pressure (CPAP) in patients with OSA on sRAGE levels.

## Methods

### Design and setting of the study

The present study is an ancillary study of the Epigenetics Modification in Obstructive Sleep Apnoea (EPIOSA) study (ClinicalTrials.gov identifier: NCT02131610). The EPIOSA Study is a longitudinal cohort of consecutive subjects referred to the Sleep Clinic at the Hospital Universitario Miguel Servet due to suspected OSA between March 2013 and March 2016 [16]. The methods used are provided in more detail (see Additional file 1). Full inclusion and exclusion criteria are shown in Additional file 1: Table S1. In brief, subjects aged 18 to 70 years and free of any additional chronic comorbid condition other than OSA or COPD were included in the cohort and visited every year at the clinic. Study procedures were carried out in accordance with the World Medical Association Declaration of Helsinki. The research protocol was approved by the Ethics and Clinical Research Committee of the Aragon Institute of Health Sciences (IRB03/2013), and informed consent was obtained from each subject.

### Clinical data, measurements, and follow-up

Demographic, anthropometric, and clinical data were obtained during recruitment and at the annual visit. Daytime somnolence was assessed using the Epworth Sleepiness Scale (ESS) [17]. Home-unattended sleep studies were performed at baseline and after one-year follow-up. Recordings were manually scored following national guidelines [18] (Additional file 1: Methods). At baseline, spirometry was performed on all participants according to American Thoracic Society (ATS)/European Respiratory Society (ERS) recommendations [19].

Subjects were categorized into five groups: (a) healthy nonsmokers (HNS); (b) asymptomatic healthy smokers with normal spirometry and no OSA (HS); (c) OSA if apnoea–hypopnoea index (AHI) was > 5 events per hour of sleep recording; (d) COPD as defined by a smoking history ≥ 10 pack-years and a postbronchodilator forced expiratory volume at first second -FEV_1_-/forced vital capacity < 0.7 after 400 μg of albuterol; and e) OSA-COPD overlap when OSA and COPD coexist in the same patient. From fasting blood samples, plasma was stored at − 80 °C for later batch analyses of sRAGE following a standard procedure (Additional file 1: Methods).

The Spanish Respiratory Society Guidelines for the management of OSA were applied [18]. Specifically, CPAP therapy was recommended if AHI ≥ 30 events/hour or AHI 5.0–30 and excessive daytime sleepiness (ESS ≥ 10) interfered with daily activities. All participants were followed at the Sleep Clinic at 3 and 12 months after the initial visit.

### Statistical analysis

Mean (and standard deviation) if normally distributed or median (and interquartile range) if not normally distributed and absolute frequency (and percentage) were computed, respectively for quantitative and qualitative data to report differences between the studied groups. The Mann–Whitney test or Fisher’s test was conveniently performed to statistically assess differences between groups. Different linear models considering the main effects of OSA, COPD and overlap on sRAGE levels but also models considering only OSA, COPD and their interaction were fitted. Different covariates were considered for the adjusted analyses, and R squared was used to measure the performance of the models in terms of explained variability. Compared to HNS, the reduction (in percentage) in the sRAGE levels was computed for each group and combination. For the cohort, as a whole and within each group studied, we used linear regression analysis to evaluate the association of COPD-related severity variables (FEV_1%_ pred) and OSA-related severity variables (AHI or men SpO2) with sRAGE circulating levels after natural log-transformation (age, sex, BMI and smoking status included as covariates). Differences between baseline and one-year follow-up sRAGE levels were studied using the Wilcoxon signed-rank test. The threshold for significance was set at 5% (alpha = 0.05), and all analyses were performed using STATA v.12.1 software (College Station, Tx, USA).

## Results

### Population

**Fig. 1:**
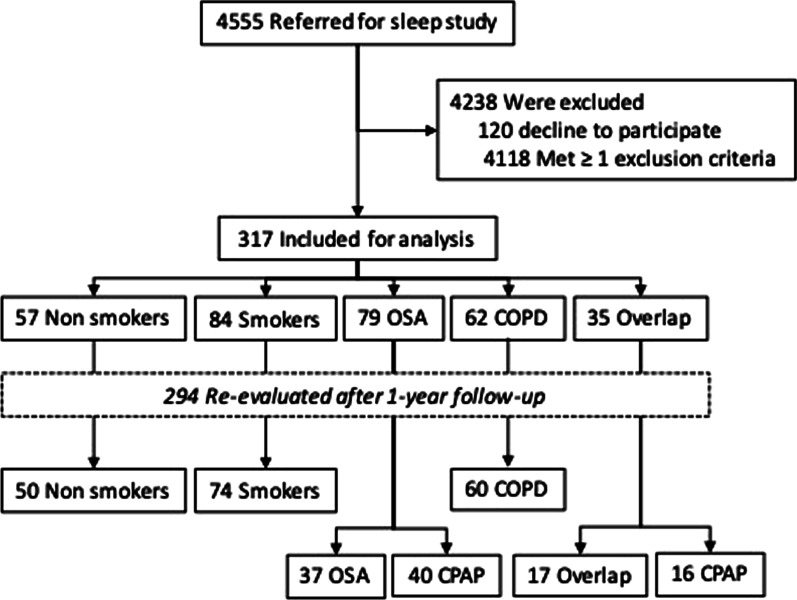
Study flowchart. Numbers of participants who were screened, assigned to a study group, and included in the analyses after 1-year follow-up according to treatment. *OSA* obstructive sleep apnoea, *COPD* chronic obstructive pulmonary disease, *CPAP* continuous positive airway pressure

A total of 317 subjects were included in the study, and 294 had also been evaluated at the one-year follow-up (Fig. 1). The demographics and clinical characteristics of the subjects at baseline are shown in Table 1. As groups, COPD and overlap patients were older and more likely to be male than heathy subjects. OSA and overlap had a higher BMI than heathy subjects or COPD patients. Patients with COPD and overlap had more pack-years than OSA and healthy smokers. Among patients with OSA and overlap, 58% and 34% respectively showed moderate-severe OSA defined as AHI > 15 with predominance of apneas and no hypopneas.

**Table 1:** Baseline clinical characteristics of participants

	Healthy nonsmokers	Smoker	OSA	COPD	Overlap
Number	57	84	79	62	35
Age, years	43.8 ± 12.0	51.8 ± 10.2	47.3 ± 9.6	60.6 ± 5.7	61.7 ± 4.7
Male, no. (%)	30 (53)	44 (52)	64 (81)	47 (68)	29 (83)
BMI, kg/m^2^	27.1 ± 4.3	26.1 ± 3.8	29.5 ± 3.5	26.3 ± 4.4	31.7 ± 5.1
Active smokers,%	0	34	18	31	27
Smoking, pack-years	0	21.3 ± 15.6	14.1 ± 17.4	41.2 ± 28.3	43.2 ± 24.7
FEV_1_, % pred	98.2 ± 11.8	91.4 ± 14.5	96.1 ± 13.6	63.5 ± 20.1	60.2 ± 19.8
AHI, events/h	2.4 ± 1.5	2.9 ± 1.4	43.7 ± 22.4	1.9 ± 2.1	29.6 ± 9.3
T90, %	1.7 ± 4.9	2.7 ± 6.6	19.1 ± 19.9	11.4 ± 17.6	29.8 ± 23.6
ICS, no. (%)	0	0	0	20 (32%)	12 (34%)
sRAGE, pg/mL	1421 (1019–1740)	1401 (1136–1731)	1148 (773–1394)	1066 (537–1281)	1047 (757–1191)

Data are presented as the mean ± sd, except for sRAGE which are presented as median (interquartile range). *HNS* healthy nonsmokers, *HS* healthy smokers, *BMI* body mass index, *FEV*_*1*_ forced expiratory volume in 1 s, *AHI* apnoea–hypopnea index, *T90%* of time with arterial oxygen saturation < 90%, ICS treatment with inhaled corticosteroides, *sRAGE* soluble receptor for advanced glycation end-products

### Plasma levels of sRAGE

At baseline, plasma sRAGE levels showed a non-normal distribution in all groups and were similar between HNS and HS. Nevertheless, median plasma sRAGE was significantly lower in OSA (1148 pg.mL^−1^), COPD (1066 pg.mL^−1^) and overlap groups (1047 pg.mL^−1^) than in healthy nonsmoker subjects (1421 pg.mL^−1^; all *p* < 0.01)(Table 1).

**Fig. 2:**
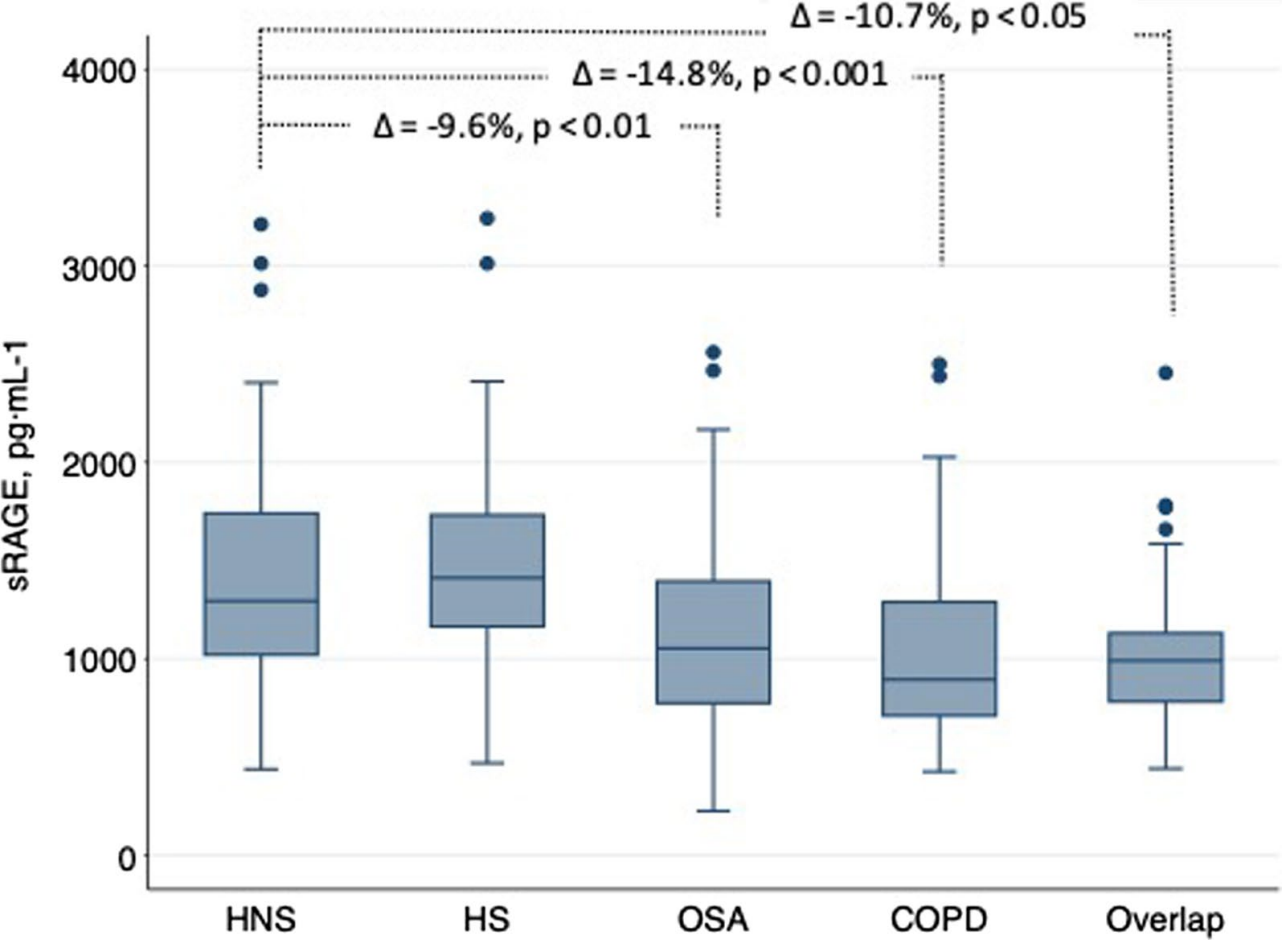
Soluble receptor for advanced glycation end-products (sRAGE) values. Median and corresponding interquartile range for each group. Reduction (in percentage) in the sRAGE levels versus the healthy nonsmoker group with the corresponding adjusted p values. *HNS* healthy nonsmokers, *HS* healthy smokers, *OSA* obstructive sleep apnoea, *COPD* chronic obstructive pulmonary disease

Adjusted regression models showed that sRAGE levels were significantly lower in OSA patients (reduction of 12.5%, *p* = 0.005), COPD patients (reduction 14.8%, *p* < 0.001) and patients with overlap (reduction 10.4%, *p* = 0.034) than in healthy nonsmokers (Table 2). There were no differences in the levels of sRAGE when comparing OSA with COPD or overlap patients. Overall, these results support the individual effect of OSA and COPD on sRAGE levels but not an additive effect (Fig. 2). In a secondary analysis, when subjects were separated based on smoking status (active versus nonactive smokers), plasma sRAGE remained significantly lower in the OSA, COPD and overlap patients compared to healthy smokers (Additional file 1: Fig. S1).

**Table 2:** Association of diagnostic groups with circulating levels of sRAGE

	Beta (SE)	Adjusted p values	Reduction of sRAGE
Intercept	2417.279 (240.523)		
OSA	− 231.915 (81.951)	0.005	− 9.6%
COPD	− 357.286 (89.676)	< 0.001	− 14.8%
Overlap syndrome	− 240.357 (82.341)	0.034	− 10.4%

### Association of sRAGE with clinical characteristics

In the cohort as a whole (Table 3), simple univariate linear regression analysis showed that plasma sRAGE was inversely related to age, male sex, BMI, pack-years of cigarette exposure, AHI and percentage of nighttime with SpO2 < 90% -T90- (all p values < 0.001) and directly related to FEV_1_% predicted (p < 0.001). However, in multivariate analysis sRAGE level remained independently related to FEV_1_ percentage predicted and inversely related to AHI and T90**.** When the analysis was restricted to subjects without COPD or OSA (n = 141), the sRAGE levels only correlated with BMI (*p* = 0.008) (Additional file 1: Table S2). In patients with OSA only (n = 79), there was an inverse relationship between sRAGE plasma levels and AHI (*p* = 0.013) (Additional file 1: Table S3), whereas in patients with COPD only (n = 62), circulating levels were independently related to FEV_1_% predicted (*p* = 0.001) (Additional file 1: Table S4). In patients with both diseases (n = 35), there was an independent relationship between the sRAGE levels and FEV_1_% (*p* = 0.013) and T90 (*p* = 0.037) as a surrogate of the degree of nocturnal hypoxemia (Additional file 1: Table S5).

**Table 3:** Univariate and multivariate association of sRAGE (log_10_) with clinical characteristics in the whole cohort

*Univariate*	Beta	SE	t test	*p* Value
Age, years	− 0.0077	0.0182	2.91	< 0.001
BMI, kg/m^2^	− 0.0206	0.0045	4.55	< 0.001
Smoking, pack-years	− 0.0032	0.0007	4.15	< 0.001
FEV_1_, % pred	0.0052	0.0009	6.01	< 0.001
AHI, events/h	− 0.0032	0.0008	4.06	< 0.001
T90, %	− 0.0078	0.0012	6.15	< 0.001
*Multivariate*				
Age, years	− 0.0047	0.0344	1.82	0.061
BMI, kg/m^2^	− 0.0129	0.0141	2.33	0.027
Smoking, pack-years	− 0.0054	0.0124	2.71	0.019
FEV_1_, % pred	0.0022	0.0005	3.16	0.002
AHI, events/h	− 0.0015	0.0005	2.63	0.012
T90, %	− 0.0039	0.0015	2.61	0.010

*BMI* body mass index, *FEV*_*1*_ forced expiratory volume in 1 s, *AHI* apnoea–hypopnoea index, *T90%* of time with arterial oxygen saturation < 90%, s*RAGE* soluble receptor for advanced glycation end-products

### Follow-up

**Fig. 3:**
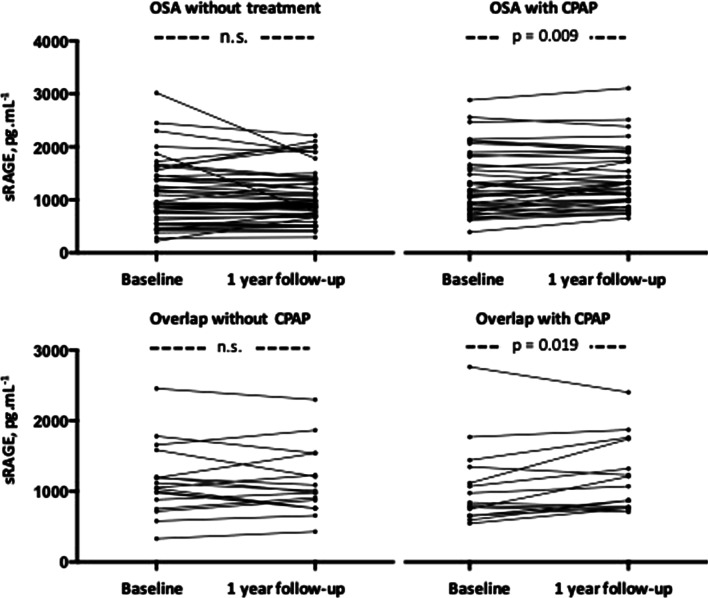
Individual values of sRAGE at baseline and at the end of the first year of follow-up. *OSA* obstructive sleep apnoea, *CPAP* continuous positive airway pressure

One-year follow-up was completed by 294 participants (92.7%). Before the scheduled visit to one year, five patients died (1 HS, 1 OSA, 2 COPD and 1 with overlap), 16 subjects declined a re-examination (7 HNS, 8 HS, 1 OSA and 1 with overlap), and two could not be reached (1 HNS and 1 HS). The mean time of CPAP use among patients with OSA or with overlap syndrome was 5.8 h per night (range 3.1–9.4) and 4.8 (range 2.6–7.7), respectively. In healthy nonsmokers, healthy smokers, and COPD patients, there were no changes in sRAGE levels (Additional file 1: Table S6 and Additional file 1: Fig. S2). There were also no changes in patients with OSA or overlap syndrome not treated with CPAP. However, in patients receiving CPAP, sRAGE levels increased significantly both in patients with OSA and in patients with overlap syndrome (p = 0.009 and p = 0.019, respectively) (Additional file 1: Table S6 and Fig. 3). The percentage change in sRAGE was not related to the baseline severity of COPD or OSA assessed by % predicted of FEV_1_ and AHI, respectively, or to the hours of CPAP use (data not shown). Excluding patients receiving treatment with inhaled steroids, the results did not change.

## Discussion

The present study shows that plasma concentrations of the anti-inflammatory molecule sRAGE are reduced in patients with COPD and in patients with OSA. Furthermore, plasma sRAGE levels are not significantly reduced in overlap syndrome with respect to COPD or OSA. Moreover, to the best of our knowledge, this is the first study that has investigated the effect of CPAP on plasma levels of sRAGE.

The receptor for advanced glycation end products is found on endothelial and inflammatory cell surfaces and binds to circulating advanced glycation end products, activating a proinflammatory protein cascade that contributes to systemic oxidative stress and inflammation. sRAGE is the soluble isoform of RAGE and acts as a protective decoy by buffering inflammatory ligands, thus decreasing inflammatory injury. Therefore, low levels of sRAGE are a biomarker of deficient inflammatory control. In addition, activation of nuclear factor-kB is an absolute requirement for both IL-6 and IL-8 via RAGE [20]. sRAGE concentrations remain relatively stable over time in community studies [21]. In both OSA and COPD, NF-kB is activated, and consequently, sRAGE may be related to the activation and maintenance of this factor and for extension in the maintenance of inflammation in COPD and OSA [22, 23]. We did not find an overlap effect on sRAGE levels. This finding could suggest that the pathways involved in the decrease in plasma sRAGE are the same for both diseases, producing a ceiling effect. The associations described in the present study are in agreement with previous reports that indicate reduced plasma sRAGE levels related to COPD and OSA [24, 25]. The decrease in COPD is related to disease severity in concordance with the ECLIPSE cohort, where levels of circulatory sRAGE were significantly reduced in accordance with advanced GOLD stage [15]. We also found an independent relationship between AHI and sRAGE in OSA, in agreement with another study [25]. In patients with only OSA or only COPD, robust signals of tissue hypoxia, such as T90 or mean nocturnal SaO2, showed no relationship with plasma levels of sRAGE. This suggests an independent role of the obstructive events “per se” and flow limitation, respectively, with the role of nocturnal hypoxemia as a secondary intermediate factor to explain the low levels of sRAGE in these diseases.

Coexistence of OSA likely still puts COPD patients at increased risk of cardiovascular death through mechanisms like repetitive episodes of upper airway obstruction and negative intrathoracic pressure at nighttime translating into increased sympathetic nerve activity at daytime probably independently from intermittent hypoxia at nighttime. The results of this study indicate that both COPD and OSA hypoxia-related diseases promote a decline in anti-inflammatory patient conditions. Moreover, the presence of these two diseases manifesting together does not have an additive effect, so the increased cardiovascular morbidity in overlap syndrome would not be explained for an increased anti-inflammatory decline for the combination of these two hypoxia-related diseases. A similar ceiling effect has been previously postulated for the influence of OSA and obesity on metabolic hormones [26]. In our study, other proinflammatory biomarkers were not evaluated; therefore, we could not establish their relationship with sRAGE levels at baseline or over time. In COPD, there is consensus in considering this disease to be associated with systemic inflammation [1]. However, in OSA, our data and those of other researchers indicate that circulating inflammatory biomarkers are not elevated and do not change with CPAP treatment [27–29]. Of interest in this study has been to find a clear reduction of a circulating anti-inflammatory marker such as sRAGE in patients with OSA, with or without associated COPD and to verify how it increased after one year of treatment with CPAP. This finding should be checked with further studies, but it raises the hypothesis of a new intermediate mechanism to explain the relationship between OSA and cardiovascular diseases and the benefit of CPAP to reduce the risk of morbidity and mortality in these patients. Interestingly, in patients with subclinical atherosclerosis, low levels of sRAGE were associated with a high intima-media thickness and carotid atheroma plaques [30]. We have previously demonstrated that in OSA patients (with or without coexisting COPD), subclinical atherosclerosis is highly prevalent and that there is an accelerating ageing process [31]. We also have reported that CPAP slows this phenomenon [32], so the long-term benefit of CPAP therapy in patients with OSA could in part be explained by increasing sRAGE with CPAP therapy. However, the biological effect of changes in sRAGE over time have not yet been well studied.

Our study has limitations. First, this study is not a randomized trial; therefore, a cause-effect relationship of the modification of sRAGE levels in relation to CPAP treatment cannot be established. Nevertheless, we studied relatively well-matched subject groups and provided one-year follow-up data on OSA treatment effects. Second, we used home-based respiratory devices rather than the gold standard inpatient overnight polysomnography. However, this approach is well established in clinical care [33–35]. Third, all patients with overlap were treated with CPAP and it is possible that the effect of BiPAP on sRAGE levels was different in subjects with associated hypercapnia. This issue should be evaluated in future studies. Finally, our sample population was drawn from hospital-based respiratory clinics; hence, we cannot extend our results to the general patient population.

## Conclusions

The present study identifies that plasma sRAGE is reduced in patients with COPD and in patients with OSA in a dose–effect relationship according to the severity of both entities. Moreover, overlap of COPD and OSA does not lead to an additive effect. Nocturnal hypoxemia does not explain these changes in either of the two entities. Effective treatment by CPAP of subjects with obstructive apnoeas (with or without associated COPD) increases the level of sRAGE, while in healthy subjects and COPD without OSA, these levels do not change over time. Future longitudinal studies are needed to ascertain whether sRAGE has utility in predicting comorbidities and mortality in OSA, COPD and OSA/COPD overlap syndrome.

## Supplementary Information

**Additional file 1.** Additional methods, figures, tables. **Table S1**. Inclusion and exclusion criteria. **Table S2.** Univariate association of sRAGE (log_10_) with clinical characteristics in healthy subjects, nonsmokers, and smokers (n = 141). **Table S3.** Multivariate association of sRAGE (log10) with clinical characteristics in patients with only OSA (n = 79). **Table S4.** Multivariate association of sRAGE (log_10_) with clinical characteristics in patients with COPD (n = 62). **Table S5.** Multivariate association of sRAGE (log_10_) with clinical characteristics in patients with OSA-COPD overlap (n = 35). **Table S6.** Median and interquartile range of sRAGE values at baseline and at 1-year follow-up according with studied groups. **Figure S1. **Soluble receptor for advanced glycation end-products (sRAGE) values. Median and corresponding interquartile range for each diagnostic group separated based on smoking status at baseline. HS: healthy smokers; OSA: obstructive sleep apnoea; COPD: chronic obstructive pulmonary disease. **Figure S2. **Individual changes in sRAGE among healthy nonsmokers, smokers and patients with COPD from baseline to one-year follow-up.

## Data Availability

The datasets used and analysed during the current study are available from the corresponding author on reasonable request.

## Abbreviations

AGE: Advanced glycation end products
AHI: Apnoea–hypopnoea index
ATS: American Thoracic Society
BMI: Body mass index
COPD: Chronic obstructive pulmonary disease
CPAP: Continuous positive airway pressure
EPIOSA: Epigenetic Modification in Obstructive Sleep Apnoea
ERS: European Respiratory Society
ESS: Epworth Sleepiness Scale
FEV1: Forced expiratory volume in the first second
HNS: Heathy nonsmokers
NS: Nonsmokers
OSA: Obstructive sleep apnoea
SpO2: Pulse oxygen saturation
sRAGE: Soluble receptor for advanced glycation end-products
T90: Percentage of nighttime with SpO2 < 90
