# Supplementary material for: Soluble RAGE in COPD, with or without coexisting obstructive sleep apnoea
Source: Respir Res. 2022 Jun 21;23:163. doi: 10.1186/s12931-022-02092-9 (PMC9210762; doi:10.1186/s12931-022-02092-9)
Supplement: Supplementary file 1 — Additional file 1. Additional methods, figures, tables. Table S1. Inclusion and exclusion criteria. Table S2. Univariate association of sRAGE (log10) with clinical characteristics in healthy subjects, nonsmokers, and smokers (n = 141). Table S3. Multivariate association of sRAGE (log10) with clinical characteristics in patients with only OSA (n = 79). Table S4. Multivariate association of sRAGE (log10) with clinical characteristics in patients with COPD (n = 62). Table S5. Multivariate association of sRAGE (log10) with clinical characteristics in patients with OSA-COPD overlap (n = 35). Table S6. Median and interquartile range of sRAGE values at baseline and at 1-year follow-up according with studied groups. Figure S1. Soluble receptor for advanced glycation end-products (sRAGE) values. Median and corresponding interquartile range for each diagnostic group separated based on smoking status at baseline. HS: healthy smokers; OSA: obstructive sleep apnoea; COPD: chronic obstructive pulmonary disease. Figure S2. Individual changes in sRAGE among healthy nonsmokers, smokers and patients with COPD from baseline to one-year follow-up. [file 12931_2022_2092_MOESM1_ESM.docx]

**ADDITIONAL MATERIAL**

**Soluble RAGE in COPD, with or without coexisting obstructive sleep apnoea**

Marta Marin-Oto ^1^, Fernando Santamaría-Martos ^2^, Ivan Benitez ^2^, David Sanz-Rubio ^1^, Ana L. Simon ^1^, Marta Forner ^1^, Pablo Cubero ^1^, Ana V. Gil ^1^, Manuel Sanchez-delaTorre ^2,3^, Ferran Barbe ^2,3^, and José M. Marin ^1,3,4^*

^1^Translational Research Unit, Aragón Health Research Institute, Zaragoza, Spain.

^2^Respiratory Department, Hospital Arnau de Vilanova, IRB-Lleida, Lleida, Spain.

**^3^** CIBER Enfermedades Respiratorias, Instituto Salud Carlos III, Madrid, Spain.

**^4^**Respiratory Service, Hospital Universitario Miguel Servet & Department of Medicine, University of Zaragoza, both at Zaragoza, Spain.

**Additional file 1**

**Methods:**

**Study subjects.**

We included subjects of 18 to 70 years of age consecutively recruited from March 2013 until March 2016 among those referred for sleep study due to suspected OSA at the Hospital Universitario Miguel Servet Respiratory Unit in Zaragoza, Spain. The inclusion and exclusion criteria are shown in **Table S1**. Demographic, anthropometric, and clinical data were obtained at recruitment using specific questionnaires and standard measurements by investigators. Height and weight were measured, and body mass index (BMI) was calculated as weight in kilograms divided by height in metres squared. The diagnosis of prevalent chronic diseases was established according to the clinical history and use of specific medications obtained from the database of the Pharmacy Department of Health Services of the province of Aragon, Spain, as described previously[1].

A sample size estimation was performed based on previous data that demonstrated clinically relevant effects with effective CPAP therapy. In a randomized controlled trial, Kohler et al demonstrated that CPAP withdrawal in 20 patients with severe OSA is associated with impaired vascular endothelial dysfunction and increased urinary catecholamines, blood pressure, and heart rate compared with 20 patients with severe OSA who continued CPAP therapy[2]. Based on these assumptions, power calculation indicated that at least 20 patients treated with CPAP and 20 non-OSA control subjects will be required to obtain differences among inflammatory biomarkers after effective OSA therapy. No previous data are available to assess the effectiveness of CPAP on circulating biomarkers in patients with OSA/COPD overlap.

**Sleep studies**.

Sleep tests were conducted at home with the use of a validated portable cardiorespiratory polygraph (BITMED NGP140 (Meditel Ingeniería Médica, Zaragoza, Spain) [3]. Subjects wore the device for a single night in their own homes and returned it the next morning. The montage included airflow measured by nasal cannula, thoracic-abdominal movement by inductance plethysmography, finger pulse oximetry and body position. Trained personnel manually scored the polygraph data in accordance with American Academy of Sleep Medicine guidelines[4]. Apnoeas were defined as a >90% decrease from baseline in airflow lasting for at least 10 seconds and were classified as central if there was no respiratory effort noted on plethysmography bands and as obstructive if respiratory effort was present during apnoea. Hypopneas were defined as a >50% decrease from baseline in flow associated with an oxygen desaturation of 4% or higher. The apnoea-hypopnoea index (AHI) was defined as the number of apnoeas plus hypopneas per hour of recording, and CT90 is considered a surrogate of hypoxaemia severity and was defined as the percentage of recording time with SaO_2_ less than 90%. Those tests in which the patients claimed to sleep less than 4 hours or in which there were less than 5 hours of nocturnal recording were repeated. Subjects diagnosed with OSA without concomitant COPD and planned for CPAP treatment had a CPAP titration study with an autoCPAP device (REMstar Auto CPAP, Philips Respironics, USA) following a validated protocol[5]. In patients with OSA and concomitant COPD who required a second sleep study to titrate CPAP, we performed a full polysomnographic study as previously described [6]. Adherence to CPAP was assessed by the time counter on the device, from the start of treatment to the end of the follow-up. Adherence was defined as CPAP use for an average of 4 h per night on ≥ 70% of the nights of use. A mean daily use of more than 4 hours per day is required to maintain CPAP prescription.

**Plasma levels of sRAGE**

A single blood sample was obtained from an antecubital vein in all participants after the completion of the nocturnal sleep laboratory recording. Fasting blood samples were collected in vacutainers with EDTA and placed on ice immediately. For assessment of sRAGE levels, plasma was prepared by centrifugation for 15 min at 3000 rpm and stored at -80°C. The plasma concentration of sRAGE was determined using a commercially available enzyme-linked immunoassay (Human RAGE Quantikina ELISA, R&D Systems, Minneapolis, MN) according to the manufacturer’s instructions. This ELISA measures the total sRAGE pool in serum and plasma samples using a solid phase sandwich ELISA. The intra- and interassay coefficients of variation were 5.7% and 7.7% for sRAGE, respectively. All samples were processed in the same manner. Measurements were always performed in duplicate, and mean values were used for analysis.

**References.**

1. Villar I, Izuel M, Carrizo S, Vicente E, Marin JM. Medication adherence and persistence in severe obstructive sleep apnoea. Sleep 2009; 32:623-8.
2. Kohler M, Stoewhas AC, Ayers L, et al. Effects of continuous positive airway pressure therapy withdrawal in patients with obstructive sleep apnoea: randomized controlled trial. Am J Respir Crit Care Med 2011; 184:1192–1199
3. [Candela A](http://www.ncbi.nlm.nih.gov/pubmed/?term=Candela%20A%5BAuthor%5D&cauthor=true&cauthor_uid=15718000), [Hernández L](http://www.ncbi.nlm.nih.gov/pubmed/?term=Hern%C3%A1ndez%20L%5BAuthor%5D&cauthor=true&cauthor_uid=15718000), [Asensio S](http://www.ncbi.nlm.nih.gov/pubmed/?term=Asensio%20S%5BAuthor%5D&cauthor=true&cauthor_uid=15718000), et al. Validation of a respiratory polygraphy system in the diagnosis of sleep apnoea syndrome. [Arch bronconeumol.](http://www.ncbi.nlm.nih.gov/pubmed/?term=(respiratory+polygraphy%5BTitle%2FAbstract%5D)+AND+Bitmed) 2005; 4:71-7
4. Iber C, Ancoli-Israel S, Chesson A, American Academy of Sleep Medicine. The AASM manual for the scoring of sleep and associated events: rules, terminology and technical specifications, 2nd ed. Westchester, IL: American Academy of Sleep Medicine; 2007.
5. Masa JF, Jimenez A, Duran J, et al. Alternative methods of titrating continuous positive airway pressure: a large multicentre study. Am J Respir Crit Care Med. 2004; 170:1218-24.
6. Marin JM, Carrizo SJ, Vicente E, et al. Long-term cardiovascular outcomes in men with obstructive sleep apnoea-hypopnoea with or without treatment with continuous positive airway pressure: an observational study. Lancet 2005; 365:1046-53.

**Additional Tables and Figures**

**Tables:**

| **Table S1. Inclusion and exclusion criteria.** | |
| --- | --- |
| **Inclusion Criteria** | **Exclusion Criteria** |
| - Age 18 to 70 - Subject is capable of giving informed consent | - Alcohol use of > 3 beverages/week - Body mass index ≥ 35 kg/m^2^ - Chronic metabolic, neurologic, other non-COPD, renal, haematologic, gastrointestinal or genital-urinary disorders - Known hypertension or blood pressure > 140/90 mmHg - Present or past cardiovascular disorders - Chronic inflammatory disorders - Active infection or recent infection (< 3 months) - Malignancy - Dislipemia or statins use - Surgery within the previous 3 months - Pregnancy or likely to become pregnant - Atopy, nasal allergy, polyps - Previous therapy for OSA - Coexistence of other than OSA sleep disorders - Regular use of use of aspirin or other anti-inflammatory agents - Use of supplementary oxigen |

| **Table S2. Univariate association of sRAGE (log_10_) with clinical characteristics in healthy subjects, nonsmokers, and smokers (n = 141)** | | | | |
| --- | --- | --- | --- | --- |
|  | **Beta** | **SE** | **t test** | **p value** |
| Age | -0.0025 | 0.024 | 1.04 | 0.301 |
| Sex (male) | 0.1072 | 0.0892 | 1.26 | 0.209 |
| BMI | -0.0324 | 0.0078 | 2.52 | 0.008 |
| Active smoker | 0.1148 | 0.0654 | 1.75 | 0.082 |
| Pack-yrs | -0.0008 | 0.0013 | 0.67 | 0.505 |
| Post-FEV_1%_ pred | -0.0014 | 0.0020 | 0.65 | 0.506 |
| FEV_1_/FVC | -0.0020 | 0.0031 | 0.65 | 0.518 |
| AHI | -0.0098 | 0.0092 | 1.07 | 0.287 |
| T90 | -0.0067 | 0.0032 | 1.99 | 0.054 |
| sRAGE: soluble receptor for advanced glycation end-products; BMI: body mass index; post-BD FEV_1_ postbronchodilator forced expiratory volume in 1 s; FVC: forced vital capacity; AHI: apnoea–hypopnea index; T90: percent of time with arterial oxygen saturation <90% | | | | |

| **Table S3.**  **Multivariate association of sRAGE (log10) with clinical characteristics in patients with only OSA (n = 79)** | | | | |
| --- | --- | --- | --- | --- |
|  | **Beta** | **SE** | **t test** | **p value** |
| Age | -0.0131 | 0.0061 | 1.66 | 0.101 |
| Sex (male) | 0.2777 | 0.1423 | 1.97 | 0.053 |
| BMI | -0.0049 | 0.0078 | 0.66 | 0.511 |
| Active smoker | -0.0588 | 0.1343 | 0.44 | 0.661 |
| Pack-yrs | -0.0023 | 0.0034 | 1.75 | 0.085 |
| AHI | -0.0068 | 0.0027 | 2.53 | 0.013 |
| T90 | -0.0042 | 0.0032 | 1.82 | 0.071 |
| sRAGE: soluble receptor for advanced glycation end-products; BMI: body mass index; AHI: apnoea–hypopnea index; T90: percent of time with arterial oxygen saturation <90% | | | | |

| **Table S4.**   **Multivariate association of sRAGE (log_10_) with clinical characteristics in patients with COPD (n = 62)** | | | | |
| --- | --- | --- | --- | --- |
|  | **Beta** | **SE** | **t test** | **p value** |
| Age | -0.0087 | 0.0093 | 0.94 | 0.352 |
| Sex (male) | 0.2207 | 0.1328 | 1.64 | 0.109 |
| BMI | -0.0139 | 0.0176 | 0.79 | 0.432 |
| Active smoker | -0.0256 | 0.1187 | 0.22 | 0.824 |
| Pack-yrs | -0.0035 | 0.0019 | 1.80 | 0.079 |
| Post-FEV1% pred | 0.0084 | 0.0023 | 3.56 | 0.001 |
| T90 | -0.0040 | 0.0028 | -1.40 | 0.160 |
| sRAGE: soluble receptor for advanced glycation end-products;; BMI: body mass index; post-BD FEV_1_ postbronchodilator forced expiratory volume in 1 s; T90: percent of time with arterial oxygen saturation <90%. | | | | |

| **Table S5.**   **Multivariate association of sRAGE (log_10_) with clinical characteristics in patients with OSA-COPD overlap (n = 35)** | | | | |
| --- | --- | --- | --- | --- |
|  | **Beta** | **SE** | **t test** | **p value** |
| Age | -0.0011 | 0.0115 | 0.07 | 0.948 |
| Sex (male) | 0.0620 | 0.1529 | 0.41 | 0.688 |
| BMI | 0.0231 | 0.0138 | 1.67 | 0.106 |
| Active smoker | 0.0206 | 0.0805 | 0.26 | 0.798 |
| Pack-yrs | -0.0035 | 0.0019 | 1.80 | 0.079 |
| Post-FEV1% pred | 0.0097 | 0.0036 | 2.66 | 0.013 |
| AHI | -0.0011 | 0.0067 | 0.17 | 0.869 |
| T90 | -0.0069 | 0.0032 | 2.18 | 0.037 |
| sRAGE: soluble receptor for advanced glycation end-products; BMI: body mass index; post-BD FEV_1_ postbronchodilator forced expiratory volume in 1 s; T90: percent of time with arterial oxygen saturation <90%. | | | | |

| **Table S6. Median and interquartile range of sRAGE values at baseline and at 1-year follow-up according with studied groups.** | | | |  |
| --- | --- | --- | --- | --- |
|  | **Baseline**  Number, median (IQR) | **1-year follow-up**  Number, median (IQR) | **p value** | |
| Healthy nonsmokers | n = 57, 1421 (1019-1740) | n = 50, 1442 (1157-1674) | 0.892 | |
| Healthy smokers | n = 84, 1401 (1136-1731) | n = 74, 1406 (1189-1862) | 0.786 | |
| OSA without CPAP | n = 37, 1140 (773-1394) | n = 37, 1102 (723-1301) | 0.345 | |
| OSA with CPAP | n = 40, 1159 (825-1757) | n = 40, 1316 (985-1757) | 0.009 | |
| COPD | n = 62, 1066 (537-1281) | n = 60, 1060 (517-1201) | 0.823 | |
| Overlap without CPAP | n = 17, 1069 (547-1289) | n = 60, 1002 (464-1191) | 0.765 | |
| Overlap with CPAP | n = 16, 1046 (534-1198) | n = 16, 1198 (571-13219 | 0.019 | |
| Data are presented as number, median (interquartile range –IQR-). sRAGE: soluble receptor for advanced glycation end-products; OSA: obstructive sleep apnoea; CPAP: continuous positive airway pressure; COPD: chronic obstructive pulmonary disease. | | | |  |

**Figures:**

**Figure S1.** Soluble receptor for advanced glycation end-products (sRAGE) values. Median and corresponding interquartile range for each diagnostic group separated based on smoking status at baseline. HS: healthy smokers; OSA: obstructive sleep apnoea; COPD: chronic obstructive pulmonary disease.

**Figure S2.** Individual changes in sRAGE among healthy nonsmokers, smokers and patients with COPD from baseline to one-year follow-up.
